# Drug Reaction, Eosinophilia, and Systemic Symptoms (DRESS) Syndrome As a Mimicker of Spinal Infection: Awareness for Spinal Surgeons

**DOI:** 10.7759/cureus.7503

**Published:** 2020-04-01

**Authors:** Riaz Mohammed, Shrijit Panikkar, Mahmoud Elmalky

**Affiliations:** 1 Spine Surgery & Orthopedics, Cardiff & Vale University Health Board, Cardiff, GBR; 2 Spine Surgery, Salford Royal NHS Foundation Trust, Salford, GBR; 3 Spine Surgery, Salford Royal Hospital NHS Foundation Trust, Salford, GBR; 4 Orthopedics, Menoufia University, Shebin Alkom, EGY

**Keywords:** spinal infections, antibiotics, drug eruptions, dress syndrome

## Abstract

Drug reaction, eosinophilia, and systemic symptoms (DRESS) syndrome is a delayed severe drug hypersensitivity (type IVb) syndrome with cutaneous eruption, hematological abnormalities, and multi-organ involvement. The wide spectrum of the disease manifestations, long-term sequelae, and high mortality rates are a clinical concern. Though not commonly reported in spinal surgery patients, the use of long-term antibiotics is a potential causative agent in spinal infections. DRESS syndrome can mimic systemic spinal infections, and clinical diagnosis requires high awareness and extreme vigilance. Prompt recognition and appropriate action can mitigate the potential poor outcomes and improve patient prognosis.

## Introduction

Drug-induced hypersensitivity syndrome (DIHS), also known as DRESS (drug reaction, eosinophilia, and systemic symptoms) syndrome, is an uncommon but delayed severe systemic hypersensitivity drug reaction [1]. Medications that are associated include, but are not limited to, anticonvulsants, antibiotics, antivirals, and antidepressants [2]. The estimated incidence of DRESS syndrome ranges from 1 in 1,000 to 1 in 10,000 drug exposures [2]. Though latency periods up to three months between drug intake and clinical presentation have been reported, the condition commonly manifests 2-6 weeks post-exposure [1]. Clinical manifestations include diffuse morbilliform rash, facial swelling, anasarca, lymphadenopathy, visceral organ involvement, and systemic signs such as pyrexia or hypotension [3-5]. Hematological abnormalities commonly associated are eosinophilia and atypical lymphocytosis.

Recognizing this syndrome and early management is of importance, as the mortality rate of DRESS syndrome has been reported to be up to 10% [4]. The syndrome is not well reported in patients with spinal pathology and hence needs reporting. We present two spinal cases, differing in clinical presentation, treated at our unit who developed DRESS syndrome. Both cases highlight the importance of recognizing the pathology to avoid the complications that might happen while treating spinal infections.

## Case presentation

The following two cases were managed under our care to illustrate the clinical pathology.

Case 1

**Figure 1:**
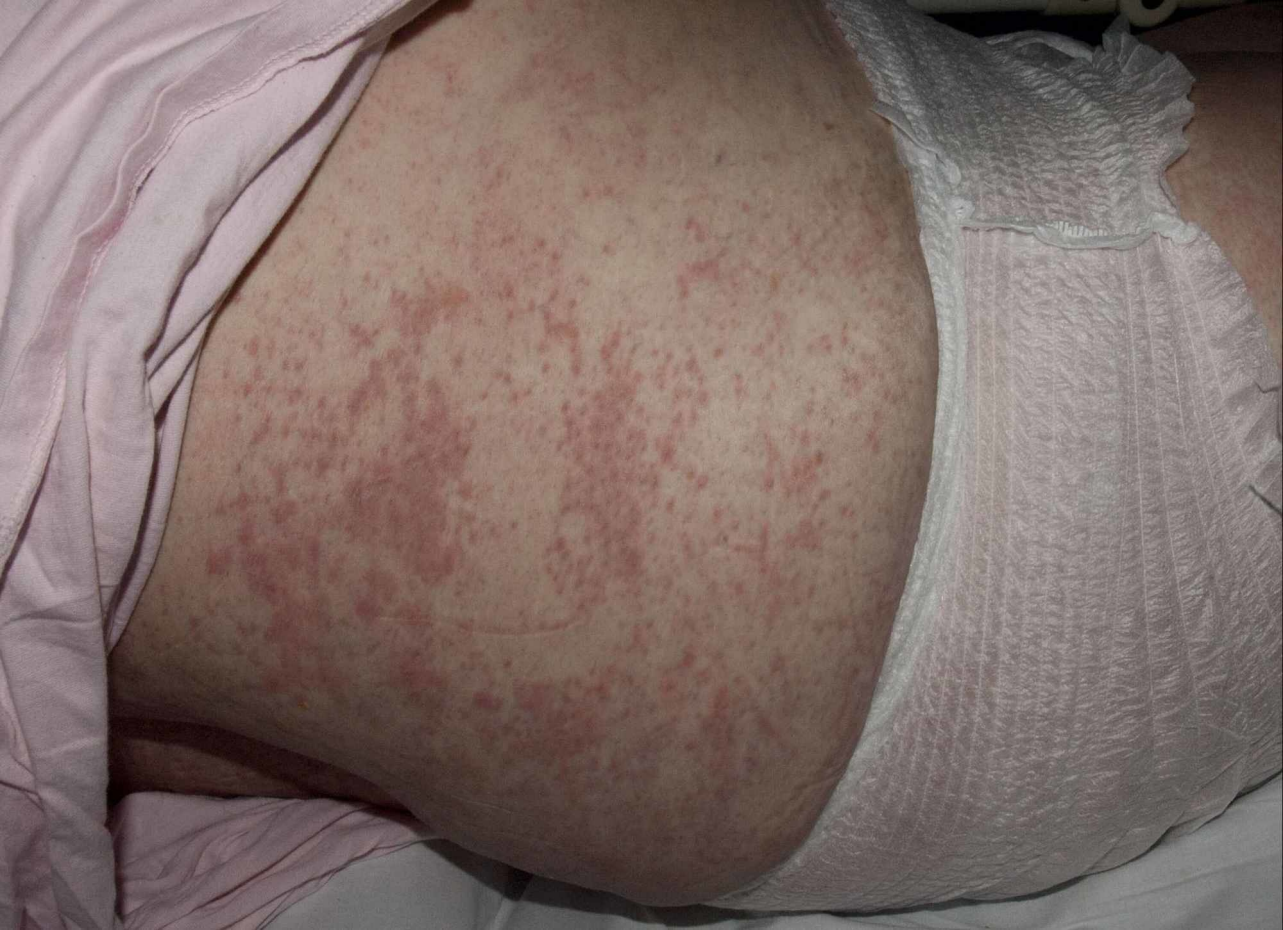
Morbilliform rash on the trunk in case 1

**Figure 2:**
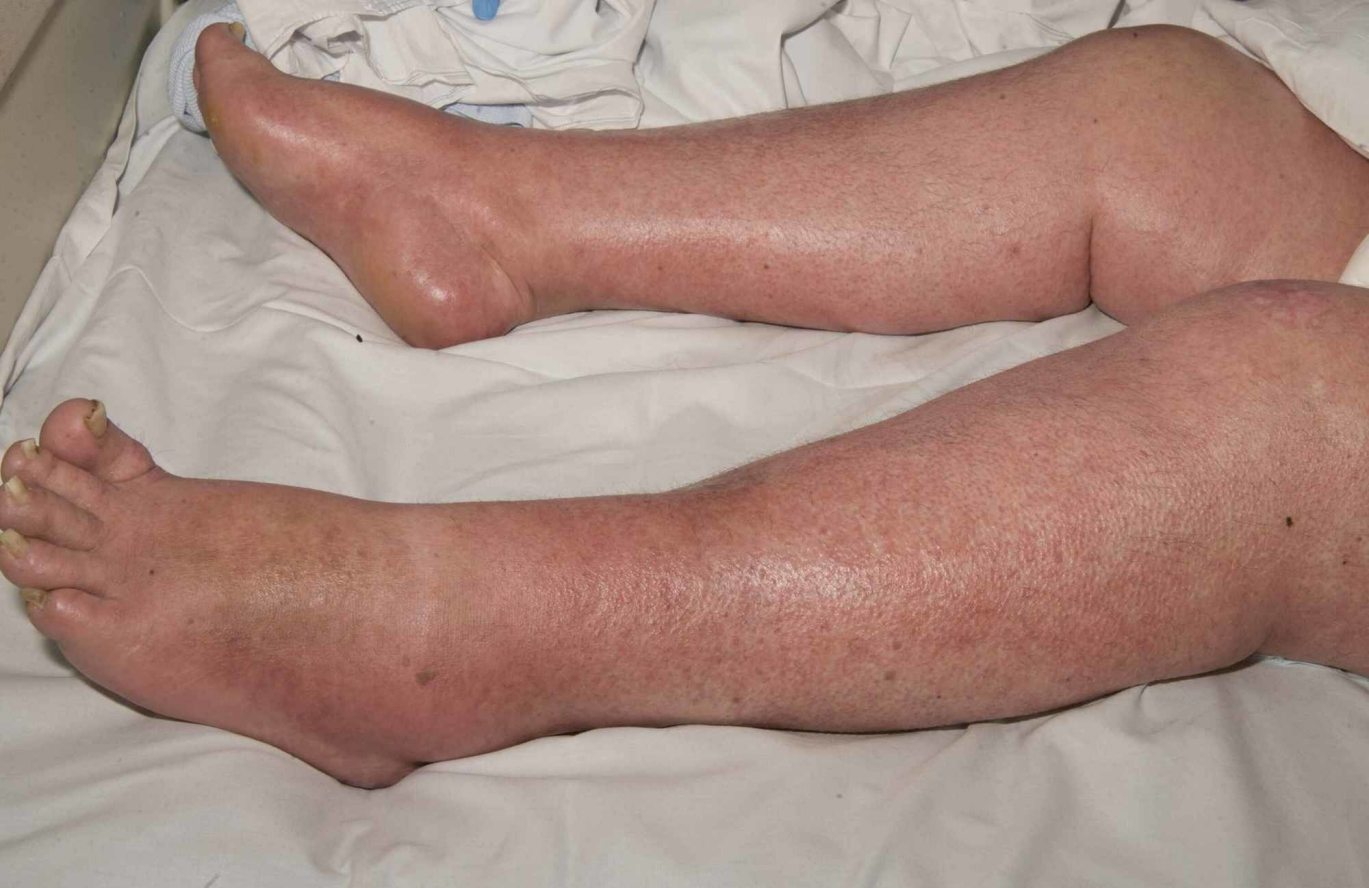
Case 1 depicting morbilliform rash on lower limbs

A 58-year-old lady presenting with back pain and leg weakness was found to have metastatic spinal cord compression. A primary tumor was suspected to be from either the pancreas or upper gastrointestinal tract. She had multi-disciplinary input for this condition and underwent thoracolumbar posterior decompression and instrumented stabilization procedure in May 2019. Unfortunately, she had wound breakdown and underwent surgical wound exploration under general anesthesia three weeks after surgery. The wound appeared to be superficially gapping, and no necrotic tissues were found; hence, it was closed with two drains. Tissue samples were positive for Staphylococcus epidermidis, and the patient was started on intravenous vancomycin and oral ciprofloxacin according to the microbiology advice. She improved clinically and the wound healed nicely; the C-reactive protein (CRP) was trending down. Three weeks later, she had high-grade pyrexia of 39.9°C. Full septic screen investigations were performed, and antibiotics were changed to intravenous vancomycin and meropenem. No foci of infection were identified from her surgical site, chest, urinary system, or blood cultures, as well as white cell total body scan. Two days later, she started to develop widespread morbilliform rash on the arms, legs, and trunk (Figures 1, 2).

**Figure 3:**
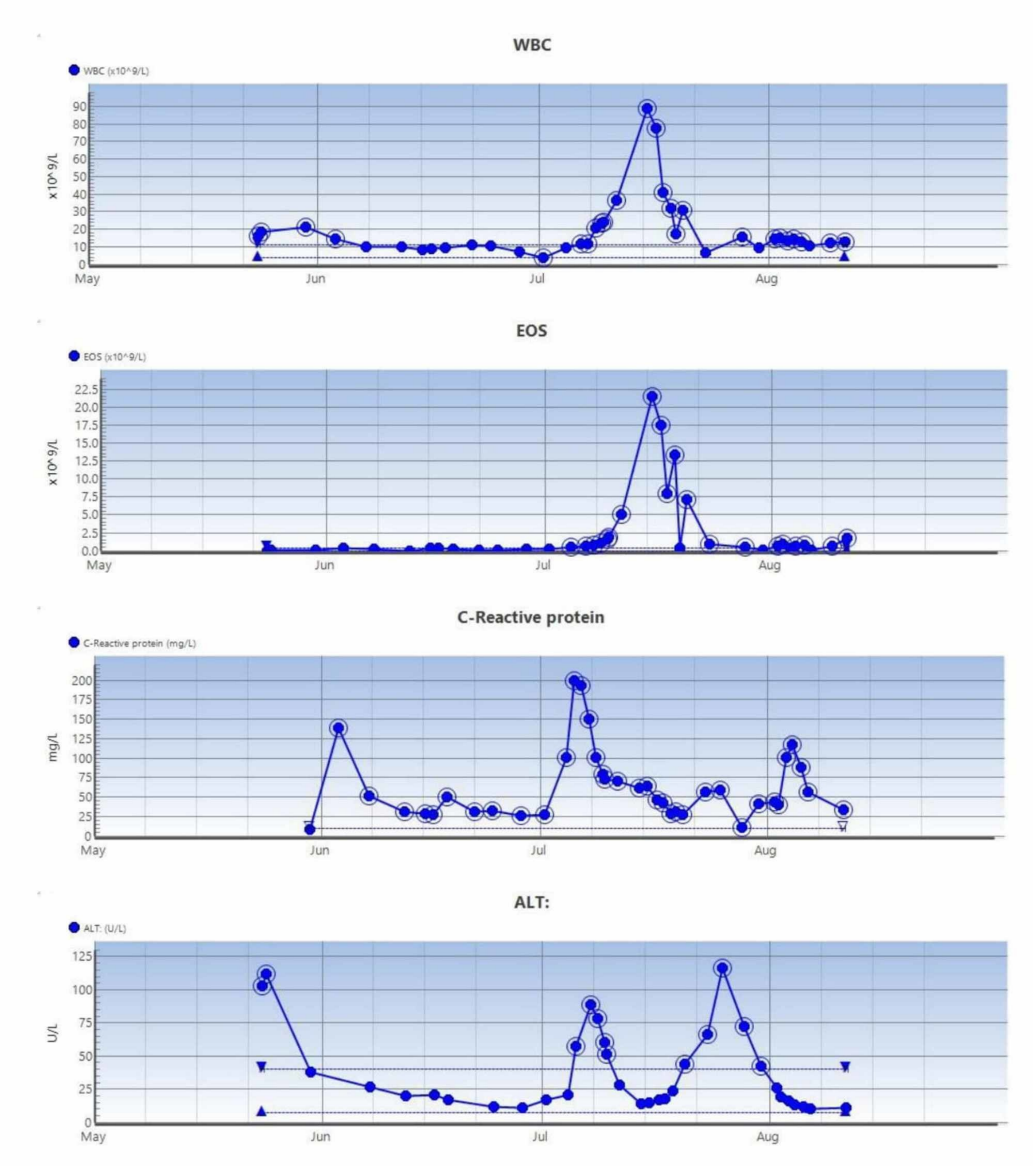
Trend of blood parameters (case 1) WBC, white blood cell count; EOS, eosinophil counts; ALT, alanine transaminase levels

There was mild facial edema, and the dermatologist suggested drug eruption due to antibiotics. Ciprofloxacin was recommenced instead of meropenem. However, her rash worsened, and uptrending levels of eosinophils along with derangement in liver function blood tests were noted. A clinical diagnosis of DRESS syndrome was made. Medication review revealed that the patient was also on morphine, naproxen, gabapentin, amitriptyline, lansoprazole, estradiol, and citalopram. Plan was made to pause all the non-essential medication, and the antibiotics were changed to tigecycline. It was felt that due to the greater risk of deep infection in the presence of metalwork, antibiotics could be continued with close monitoring as per the advice from the microbiologist. Steroids were commenced as 30 mg of prednisolone (as prescribed by the Dermatology physician), to be tapered gradually. The patient’s rash, systemic symptoms, and hematological parameters (Figure 3) improved within a few days and continued to do so till her discharge for subsequent outpatient oncological treatment.

Case 2

**Figure 4:**
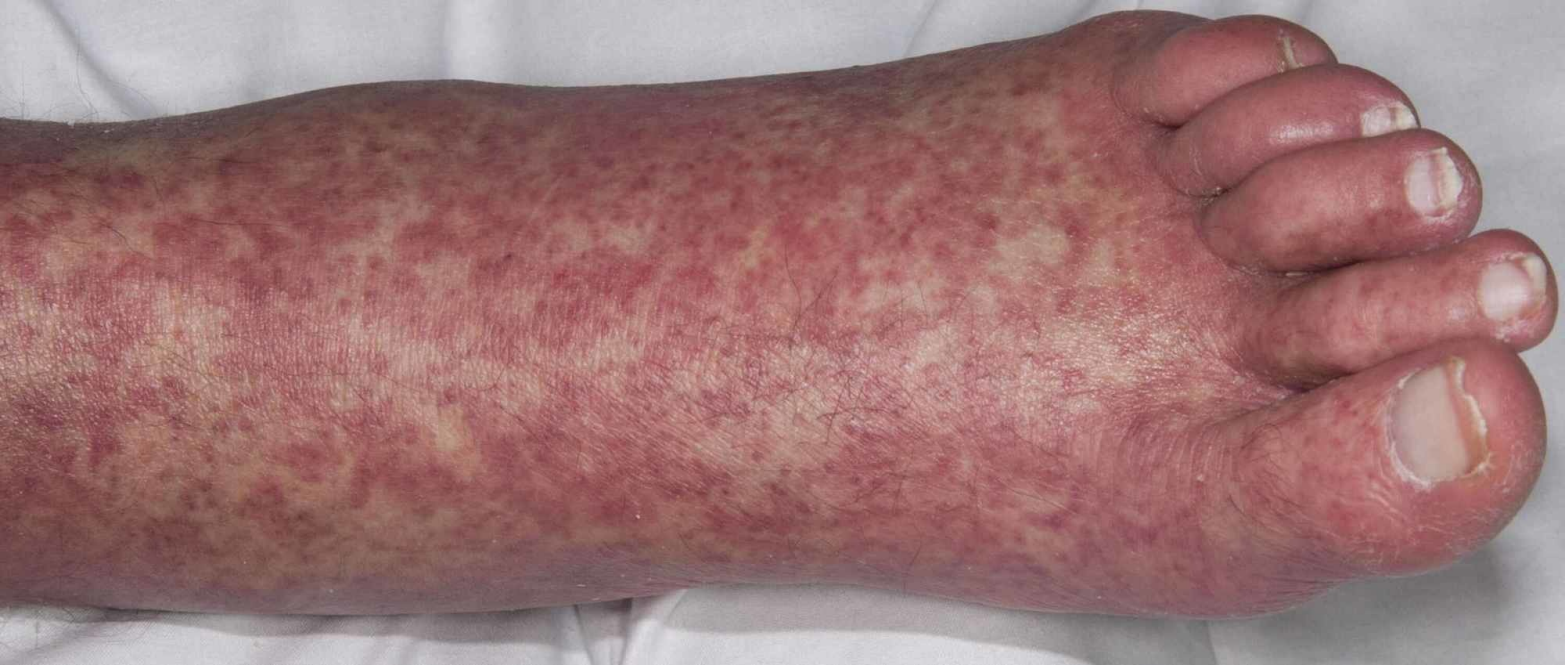
Erythematous papular rash on the feet in case 2

**Figure 5:**
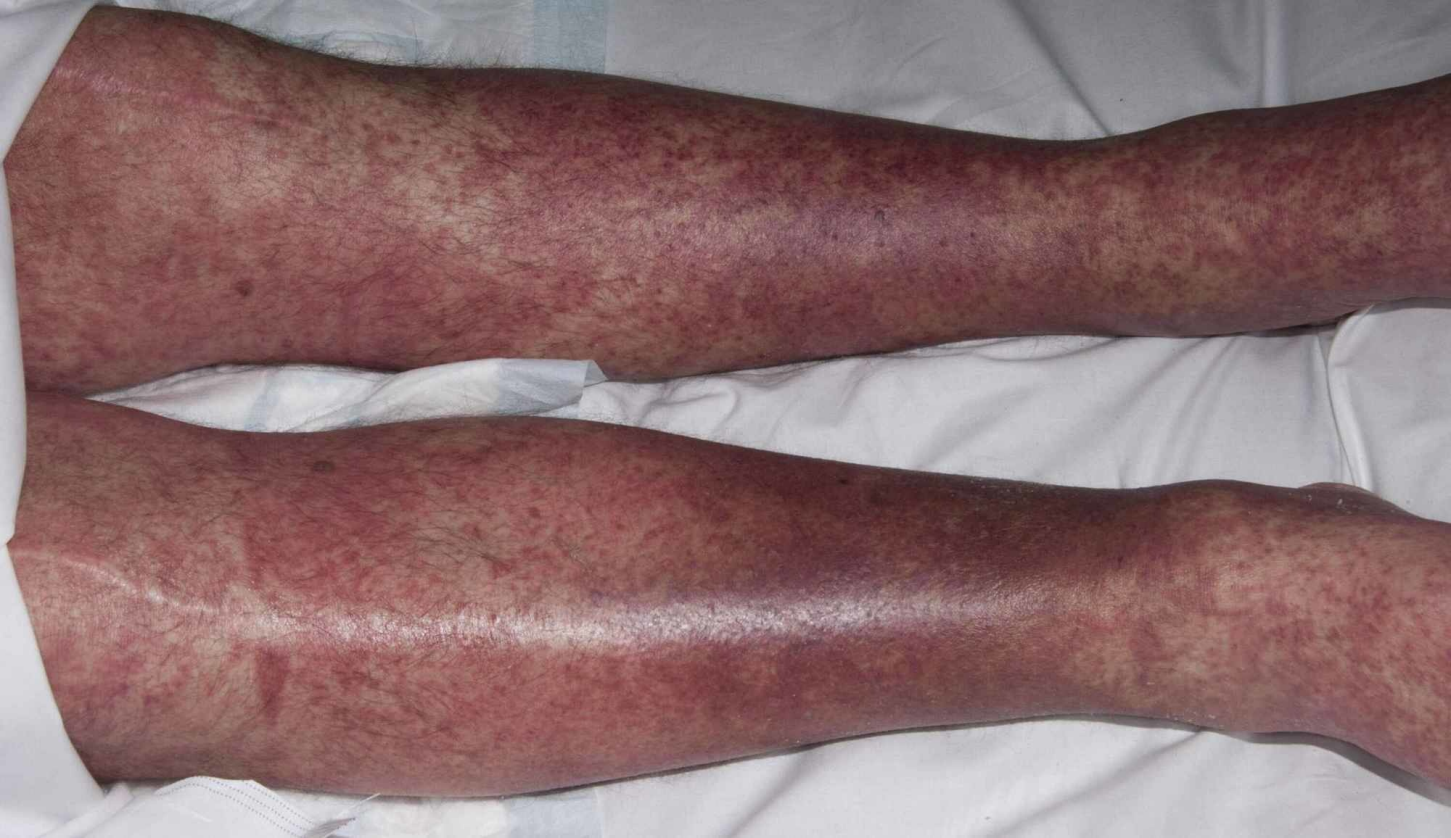
Case 2 patient showing erythematous papular rash on the legs

A 57-year-old gentleman was referred to us in November 2018 with cervical and lumbar multi-focal spondylodiscitis, which was being treated with tazocin, ertapenem, and teicoplanin for two weeks prior to transfer. He was noted to have anasarca and swelling of both hands and feet. Blood cultures and blood-borne virus’ screens were negative, but blood tests revealed elevated white cell counts (14.5 x 109/L) and a CRP of 515 mg/L. Antibiotics were changed to meropenem and vancomycin as per the microbiology specialist advice. His CRP continued to be high, and two weeks later had deranged liver function tests (LFTs) and mild hepatomegaly on ultrasound. In view of this meropenem was changed to oral ciprofloxacin. Rheumatology team suggested that his polyarthralgia and joint swellings were systemic inflammatory responses to the sepsis. Six weeks into the treatment he developed erythematous papular rash on the chest, and upper arms and legs (Figures 4, 5). Topical steroids and emollients were commenced, and vancomycin was changed to daptomycin.

**Figure 6:**
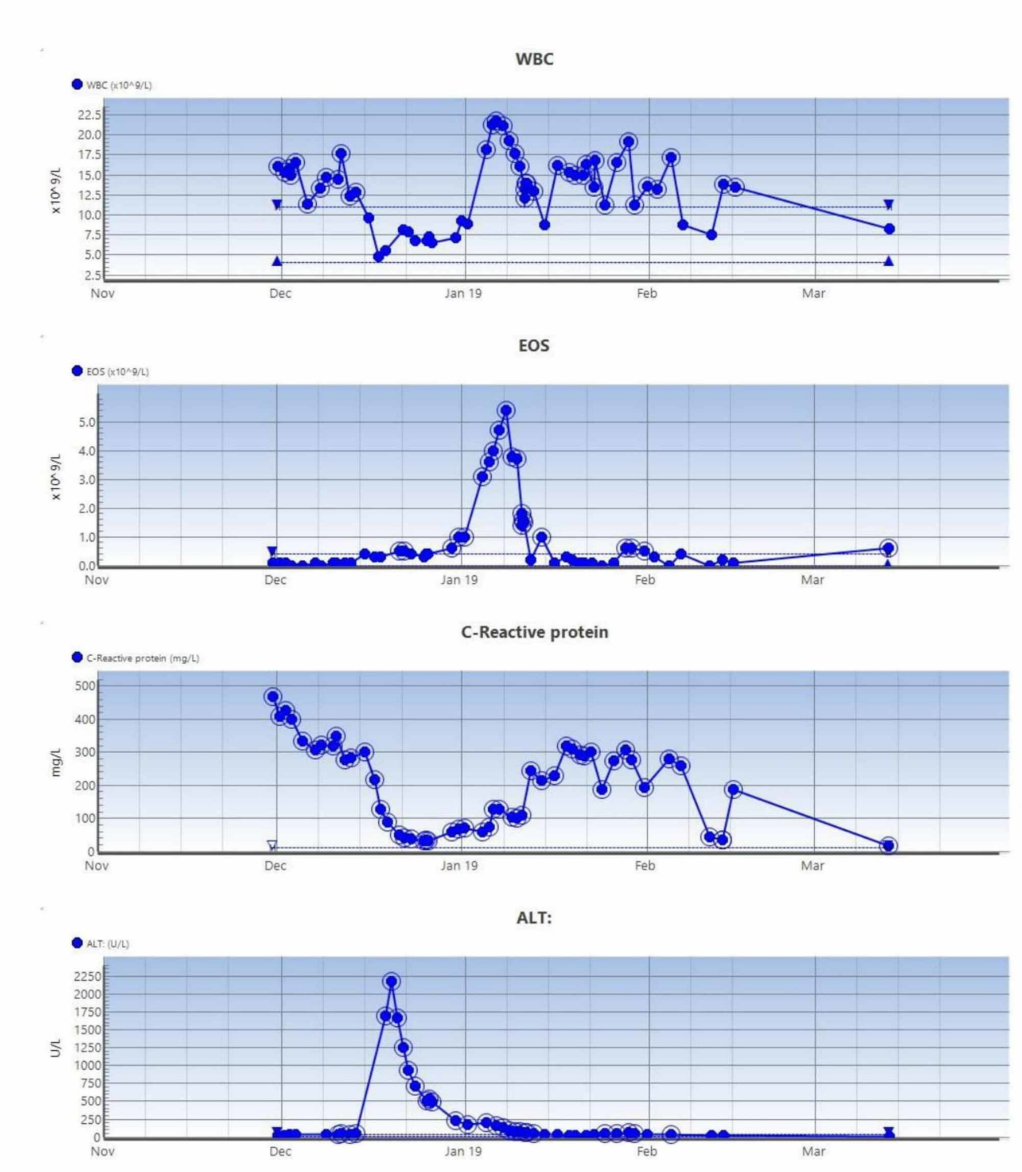
The trend of serial blood tests in case 2 WBC, white blood cell count; EOS, eosinophil counts; ALT, alanine transaminase levels

Clinically he did not have any lymphadenopathy or hepatosplenomegaly. The LFTs showed improvement, but the eosinophil count had an upward trend over the next few weeks. Dermatology review suspected either DRESS syndrome or acute generalized exanthematous pustulosis (AGEP). Cutaneous punch biopsy confirmed dermal perivascular lymphohistiocytic inflammatory cell infiltrate, red blood cell extravasation, and dermal eosinophil infiltrations. The absence of pustules on histology ruled out AGEP. Both the antibiotics were ceased, but due to deranged renal function and persistent eosinophilia, he was transferred to the intensive care unit for closer monitoring. Renal biopsy confirmed severe eosinophilic tubule-interstitial nephritis. Systemic steroid therapy was not commenced because of the possibility of a spinal infection. In view of improving renal function, liver function, eosinophil counts (Figure 6), and improvement of systemic rash, he continued to be treated conservatively. An interval MRI scan of the spine and a leukoscan suggested no active spinal infection, and he was subsequently discharged when clinically improved. He has subsequently been seen as an outpatient and continues to be under review.

## Discussion

Severe cutaneous adverse reactions (SCARs) to medications are seen as part of conditions such as Steven-Johnson syndrome, toxic epidermal necrolysis, acute AGEP, and DRESS syndrome.

Patients with DRESS syndrome can have heterogeneous clinical manifestations, which present in different timelines. A high spiking fever and rash are typically the first presentations. The rash is nonspecific morbilliform eruption indistinguishable from other allergic drug reactions but can be widespread confluent type and, in severe cases, can result in blistering. Facial edema with periorbital accentuation is also a characteristic finding as evidenced in our first patient.

Hematological anomalies such as atypical lymphocytosis and eosinophilia have been reported in up to 63% and 52%, respectively [1]. Though these hematological counts are self-limiting, findings of pancytopenia are generally associated with a poor prognosis.

The liver and kidney are the commonly afflicted visceral organs, though involvement can include almost all organ systems such as cardiac, respiratory, muscular, nervous system, pancreas, and gastrointestinal. The severity of involvement can range from a clinical spectrum of subclinical to end-stage organ failure, of which the cardiac complications can be the cause for the high mortality rate associated with DRESS syndrome.

DRESS syndrome has been reported after exposure to numerous medications including phenytoin, carbamazepine, and phenobarbital lamotrigine, oxcarbazepine, sulfasalazine, doxycycline, allopurinol, linezolid, nitrofurantoin, atorvastatin, esomeprazole, and vancomycin [2]. The pathogenesis of DRESS syndrome is not yet well understood. However, causative associations that have been linked include defects in drug metabolism, resulting in the accumulation of toxic metabolites, reactivation of viral infections that trigger the idiosyncratic reaction, and a genetic predisposition in the patient that alters the host immune response [3].

Spinal conditions that require prolonged antibiotics may be susceptible to DRESS syndrome. In both cases, vancomycin was most likely the principal agent, but other antibiotics could also be causative. There has been a recent increasing trend in vancomycin-induced DRESS reported in the literature [4]. A case series identified vancomycin as the commonest agent, causing 37.5% of DRESS over a three-year period [5].

Some of the clinical features of DRESS syndrome can also be present in patients with systemic infections. The RegiSCAR (European Registry of Severe Cutaneous Adverse Reactions to Drugs and Collection of Biological Samples) scoring system has been recently developed to delineate each of the SCARs as distinct entities. To meet the definition of DRESS, patients must have at least three of the following RegiSCAR criteria: an acute rash, fever > 38.5°C, lymphadenopathy at two sites, involvement of at least one internal organ, and abnormalities in lymphocyte and eosinophil counts. The scoring system grades suspected cases of DRESS as scores: <2 indicates no case, 2-3 indicates possible case, 4-5 indicates probable case, and >5 indicates definite case [6]. Both of our cases tallied 6 on the scoring system (Table 1).

**Table 1:** RegiSCAR scoring system for classifying cases of DRESS syndrome DRESS, drug reaction, eosinophilia, and systemic symptoms; NA, not applicable; RegiSCAR, European Registry of Severe Cutaneous Adverse Reactions to Drugs and Collection of Biological Samples

Score	−1	0	1	2	Case 1	Case 2
Fever > 38.5°C	No	Yes			0	0
Enlarged lymph nodes > 1 centimeter		No	Yes		0	0
Eosinophilia: × 10^9^/L		No	0.7–1.49	>1.5	2	2
Atypical lymphocytes		No	Yes		0	0
Skin rash > 50% body surface area		No	Yes		1	1
Skin rash suggesting DRESS	No		Yes		1	1
Biopsy suggesting DRESS	No	Yes			NA	0
Organ involvement		No	One organ	Two or more organs	2	2
Resolution > 14 days	No	Yes			0	0
Evaluation of other potential causes: >2 negative of anti-nuclear antibodies, blood culture, hepatitis A/B/C, chlamydia/mycoplasma			Yes		NA	NA
Final score					6	6

DRESS syndrome is treated with immediate cessation of the causative agent and a prolonged course of tapering dose steroids; 1-2 mg/kg in one or two divided doses of prednisolone is the standard treatment dose [7]. In case 2, our dilemma was to commence steroids in the presence of deep spinal infection, and balance steroids were not prescribed after consultation with the intensivist. Intravenous immunoglobulin, cyclosporine, cyclophosphamide, mycophenolate mofetil, and plasmapheresis have been reported to be used in steroid-resistant cases. We are not aware of many reports of DRESS syndrome in spinal patients [8]. Surgeons treating spinal infections can come across patients who develop features of DRESS syndrome, and if there is a delay in diagnosis or subsequent treatment, the outcomes for the patients can be devastating and long-standing.

## Conclusions

Spinal surgery complicated with infection or other spinal conditions such as spondylodiscitis warrants prolonged antibiotic therapy. A high index of suspicion is therefore required in patients who develop widespread rash and deranged hematological counts a few weeks after antibiotic treatment. In our patients, despite stopping the antibiotic treatment, markers for infection continued to improve. Therefore, it may be prudent to assume that many of the clinical-biochemical features seen are in fact due to DRESS syndrome rather than worsening of the existing spinal infection. Clinical dilemma arises when patients with spinal infections also develop DRESS syndrome. Multi-disciplinary input from microbiologists, dermatologists, hematologists, allied medical specialists, and intensivists is of paramount importance in managing these patients appropriately.
